# Impact of respiratory motion correction on lesion visibility and quantification in thoracic PET/MR imaging

**DOI:** 10.1371/journal.pone.0233209

**Published:** 2020-06-04

**Authors:** Marcel Gratz, Verena Ruhlmann, Lale Umutlu, Matthias Fenchel, Inki Hong, Harald H. Quick

**Affiliations:** 1 Erwin L. Hahn Institute for Magnetic Resonance Imaging, University of Duisburg Essen, Essen, Germany; 2 High Field and Hybrid MR Imaging, University of Duisburg-Essen, Essen, Germany; 3 Department of Nuclear Medicine, University Hospital Essen, Essen, Germany; 4 Department of Diagnostic and Interventional Radiology and Neuroradiology, University Hospital Essen, Essen, Germany; 5 Siemens Healthcare GmbH, Erlangen, Germany; 6 Siemens Medical Solutions Inc, Knoxville, Tennessee, United States of America; Monash University, AUSTRALIA

## Abstract

The impact of a method for MR-based respiratory motion correction of PET data on lesion visibility and quantification in patients with oncologic findings in the lung was evaluated. Twenty patients with one or more lesions in the lung were included. Hybrid imaging was performed on an integrated PET/MR system using ^18^F-FDG as radiotracer. The standard thoracic imaging protocol was extended by a free-breathing self-gated acquisition of MR data for motion modelling. PET data was acquired simultaneously in list-mode for 5-10 mins. One experienced radiologist and one experienced nuclear medicine specialist evaluated and compared the post-processed data in consensus regarding lesion visibility (scores 1–4, 4 being best), image noise levels (scores 1–3, 3 being lowest noise), SUVmean and SUVmax. Motion-corrected (MoCo) images were additionally compared with gated images. Non-motion-corrected free-breathing data served as standard of reference in this study. Motion correction generally improved lesion visibility (3.19 ± 0.63) and noise ratings (2.95 ± 0.22) compared to uncorrected (2.81 ± 0.66 and 2.95 ± 0.22, respectively) or gated PET data (2.47 ± 0.93 and 1.30 ± 0.47, respectively). Furthermore, SUVs (mean and max) were compared for all methods to estimate their respective impact on the quantification. Deviations of SUVmax were smallest between the uncorrected and the MoCo lesion data (average increase of 9.1% of MoCo SUVs), while SUVmean agreed best for gated and MoCo reconstructions (MoCo SUVs increased by 1.2%). The studied method for MR-based respiratory motion correction of PET data combines increased lesion sharpness and improved lesion activity quantification with high signal-to-noise ratio in a clinical setting. In particular, the detection of small lesions in moving organs such as the lung and liver may thus be facilitated. These advantages justify the extension of the PET/MR imaging protocol by 5–10 minutes for motion correction.

## Introduction

In recent years, hybrid positron emission tomography/magnetic resonance (PET/MR) has emerged as an important imaging modality for various pathologies and clinical questions [1–5]. Much work and focus have been put into hardware and methods development and in early clinical studies comparing PET/MR to PET/computed tomography (CT) as the well-established standard of reference in hybrid whole-body imaging [6–11]. Integrated hybrid PET/MR, where PET and MR data are acquired simultaneously rather than in a sequential fashion, proved to be a competitive and unique hybrid imaging modality for a broad range of clinical applications [2, 12]. In particular, this holds true for scans that involve highly motion-affected regions in the thorax such as lung, liver and heart and thus need the inherent accurate co-registration of a parallel image acquisition. Early studies of thoracic pathologies with either modality, PET or MR imaging alone, suggested a high diagnostic value for lesion detection, tumour staging and e.g. the estimation of chest wall infiltration [13–15], which may improve even further using a synergetic combination of both modalities.

In general, thoracic imaging demands high spatial resolution, image sharpness and high signal-to-noise ratio in the acquired images. Fast MR sequences that meet these requirements within a single breath-hold, such as the 3D volumetric interpolated breath-hold examination (VIBE) sequence, exist and are applied on a regular basis in clinical MR imaging routine. However, due to the comparably long PET acquisition times (several minutes) and limited compliance of patients that present with pathologies involving the lung, most often it is not possible to obtain diagnostic PET data that are free from respiratory motion. Thus, PET imaging in the thorax and liver is often hampered by smearing and blurring of lesions along the directions of respiratory motion, i.e. mainly head/feet and anterior/posterior. This impedes the determination of lesion size, characterization of radiotracer uptake and, consequently, the conclusion about successful treatment response. In cases of small lesion sizes and low radiotracer uptake even a reliable detection may be not possible [16, 17]. Furthermore, the introduced misregistration of the underlying static attenuation maps (*μ*-maps) can cause an over- or underestimation of activity in regions that are extensively affected by motion, such as the diaphragm [18, 19].

Due to the nonlinear nature of respiratory motion, a straightforward and rigid correction is not feasible. Nemeh et al. [20] proposed a gating method in which only the fraction of counts that belong to a specific respiratory phase were taken into account for a retrospective reconstruction. Inherently, large parts of the acquired PET coincidences are not considered for the statistics, leading to a significantly decreased signal-to-noise ratio in the final PET images. Approaches that are more computationally demanding try to use the complete data and map it to a single reference state of motion. However, extensive knowledge of the body deformation fields in three dimensions (3D) during the entire respiratory cycle needs to be derived from the obtained data. Studies using PET data alone were conducted already [21], but PET is often not sufficient to provide the necessary amount of landmarks for a robust determination of such deformation matrices [22, 23]. This particularly applies to radiotracers with comparably low uptake characteristics.

Following this concept, the use of free-breathing MR data for the generation of a body deformation model was proposed as a promising method for hybrid PET/MR modalities [24]. In first feasibility studies [25–27] multiple 2D slices were acquired using a spoiled gradient echo sequence in sagittal direction along with navigator echoes to cover the respiratory cycle with sufficient spatial and temporal resolution for the subsequent post-processing. Later approaches used a self-gated acquisition with a combined Cartesian sampling in read-out direction with a radial-like pattern in the phase encoding plane, using golden angle shift between subsequent phase steps [28] or a T1-weighted 3D radial stack-of-stars spoiled gradient echo sequence with fat suppression [29–31]. This allowed a direct derivation of the respiratory cycle and thus deformation models from the MR data without the need of further devices, such as breathing cushions, or navigator echoes. The acquired PET data is binned to a discrete number of states, which then are transformed and warped to a common respiratory reference phase.

A non-commercial prototype version of MR-based free-breathing motion correction of PET data is available for the Biograph mMR PET/MR system (Siemens Healthcare, Erlangen, Germany). Thus, encouraged by the result of technical studies such as in [32–34], the aim of this study was to evaluate the impact of respiratory motion correction in PET/MR of the thorax on the obtained image quality to facilitate and potentially improve the clinical diagnosis. The motion correction method was tested in a collective of 20 patients with oncologic findings in the lung. Results were compared to conventionally retrospectively gated and non-motion-corrected data that was derived from the same data set, as the intra-individual standard of reference for each patient.

## Materials and methods

### Patients

A total of 20 patients (13 male, 7 female) at a mean age of 64.6 ± 8.8 years with various PET-active lesions located in the thorax underwent a routine clinical imaging protocol on an integrated 3 T PET/MR system (Biograph mMR, Siemens Healthcare, Erlangen, Germany). The patient cohort was selected consecutively based on their initial diagnosis, which had to include the suspect of at least one lesion in the thoracic region, with no further specific criteria. An activity of 264.4 ± 42.6 MBq of the radiotracer ^18^F-Fluorodeoxyglucose (FDG) was injected on average 150 ± 38 mins before the actual PET/MR scan. With some patients having more than one lesion, an overall number of 43 lesions was present in the available data sets. For a detailed overview of patients and findings, refer to Table 1.

**Table 1:** Patient data overview.

#	type	gender	age yrs	activity MBq	waiting time after injection	diagnosis	lesion count
1	single bed	female	72	261	2h 48m	NSCLC	2
2	single bed	male	55	339	2h 20m	relapsed BC	1
3	single bed	female	66	195	1h 42m	ACC	1
4	single bed	female	63	275	2h 29m	NSCLC	1
5	single bed	female	53	268	3h 16m	BC	1
6	single bed	male	66	272	3h 4m	BC	1
7	single bed	male	67	250	3h 7m	Lymphadenopathy	1
8	single bed	male	71	265	1h 6m	BC	3
9	single bed	male	59	278	1h 56m	BC	2
10	single bed	female	74	245	2h 28m	Relapsed Mamma-CA	2
11	two bed	male	52	228	2h 22m	BC	5
12	two bed	male	63	328	3h 4m	BC	2
13	two bed	male	54	252	1h 57m	NSCLC	5
14	two bed	female	72	198	3h 9m	BC	3
15	two bed	male	77	305	2h 17m	BC	3
16	two bed	male	85	230	2h 22m	BC	1
17	two bed	male	65	280	2h 9m	BC	3
18	two bed	male	56	263	3h 10m	NSCLC	1
19	two bed	male	64	348	1h 51m	Adeno-CA	3
20	two bed	female	58	208	3h 38m	BC	2

NSCLC = non-small-cell lung carcinoma, BC = bronchial carcinoma, ACC = adenoid cystic carcinoma, CA = carcinoma.

The study was conducted in conformance with the Declaration of Helsinki and approved by the Ethics Commission of the Medical Faculty of the University Duisburg-Essen (study number 11–4822-BO). Written consent was obtained from all patients prior to the PET/MR examinations.

### Acquisition and post-processing

A free-breathing self-gated 3D gradient spoiled echo with a stack-stack-of-stars sampling scheme using a golden-angle increment [35] was added along with a PET list-mode acquisition to the standard PET/MR protocol right after acquisition of the localizer images and a conventional 3D Dixon VIBE sequence in end-expiratory breath-hold (TA = 0:19 min). The latter was used to calculate an attenuation map (*μ*-map) containing four classes of tissues (air, lung, muscle, and water), which is necessary to apply an appropriate MR-based attenuation correction (MRAC) in the PET reconstruction [18, 36]. The diagnostic imaging protocol further contained an axial T2 HASTE (half-Fourier-acquired single-shot turbo spin echo, acquisition time per bed position TA = 0:47 min), a coronal T2 TIRM (turbo inversion recovery magnitude, TA / bed = 2:01 min) and a 3D VIBE (pre- and post-contrast, TA / bed = 0:18 min) at five bed positions along with an axial 2D T1 FLASH (fast low angle shot sequence, in- and opposed-phase acquisition, TA = 0:36 min) across the thorax and abdomen, and a 3D T1-weighted MPRAGE sequence (magnetization prepared rapid gradient echo, TA = 5:18 min) across the head. No further external accessories, such as a respiratory belt, were used in this study.

For the first 10 out of the 20 patients, the self-gated gradient spoiled echo for motion correction was scanned as a single-bed version with 10 minutes of acquisition time and was centered at the lung, whereas for the other 10 patients, a two-bed version with approximately 1 to 2 cm overlap was used, covering an axial field-of-view from the upper mediastinum down to the complete liver. For the latter, an acquisition protocol of two times 5 minutes per bed position was set up, leading to a total acquisition time of 10 minutes. Both acquisition types were using a spatial resolution of 1.56 × 1.56 × 4.5 mm^3^ (TE = 1.32 ms, TR = 2.97 ms, fat saturation). The patients were positioned head first, supine on top of the 24-channel spine array radio-frequency (RF) coil placed on the patient table of the PET/MR system. Two additional vendor-provided multi-channel body RF arrays that were optimized for a maximum of PET transparency, i.e. with a minimal photon attenuation at 511 keV [37], were placed on the patient’s thorax for anterior MR signal reception. Altogether, the total scan duration was approximately 35 min per patient.

**Fig 1:**
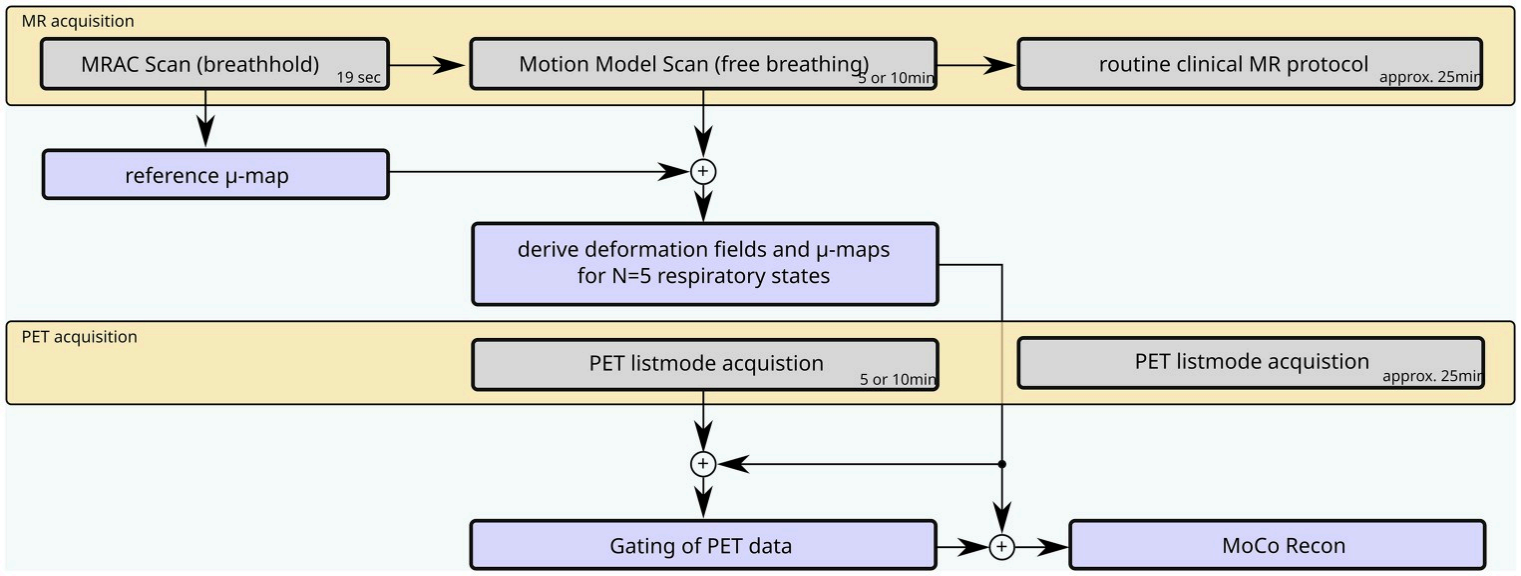
Simplified scheme of the acquisition (yellow) and post-processing (blue) for the studied motion correction method. Note that for this study the PET acquisition was subdivided into a part covering only the motion model scan and one part covering the clinical diagnostic scan for convenience and reduced interference with the routine clinical protocol itself.

For derivation of the respiratory curve the self-gating signal is used, which is derived from the central k-space portion of the added 3D radial gradient spoiled echo. Since the k-space center contains contrast information, movement of anatomy through the respective imaging plane can be directly observed by changes in the same raw MR signal that is further used for image reconstruction. The MR data were binned according to the self-gating signal into N = 5 uniformly sized phases, and each respiratory phase image was reconstructed with a non-uniform Fourier transform reconstruction after regridding and appropriate density correction. The number of five motion phases was suggested by [29] as the best trade-off between accuracy (least motion) and sufficient count statistics in each bin. Forward and backward motion fields were computed from the respiratory image phases by non-rigid registration of the images to the reference image phase in end-expiration using a variant of a diffeomorphic demons registration algorithm [38] (see Fig 1).

The conducted study was done with a predecessor prototype version of the PET reconstruction software that required an offline reconstruction (e7 tools, Siemens Molecular Imaging, Knoxville, USA) on a separate workstation. Based on the MR self-gating information, the PET list-mode data from the scanner console were histogrammed into five sinograms representing the five motion phases. An ordered subset expectation maximization (OSEM) reconstruction [39] then reconstructed a single (motion-corrected) PET image with an in-plane resolution of 4 × 4 mm^2^ using three iterations and 24 subsets. ECF (scanner calibration factor), randoms smooting and Gaussian filtering were involved in the reconstruction. Additional resolution modeling such as using the point spread function (PSF) were not used. Each update of the image is obtained by warping the current image estimate according to the deformations at all respiratory phases, projecting the warped images (utilizing a warped *μ*-map), back-projecting the residuals of the projections and warping the resulting volumes back to the reference frame. Finally the warped volumes are summed, thus providing an image which has been motion corrected to the reference frame [10, 40].

In the cases of two bed positions acquired with the motion correction protocol during the acquisition, the final PET images were merged with a linear blending algorithm in the overlap region. In addition to the studied motion correction approach (referred to as “MoCo”), conventionally gated (“gated”) and inherently motion-affected uncorrected images (“uncorrected”) were calculated for each patient using the same raw data. Gated images were reconstructed by simple OSEM reconstruction of the counts from the sinogram of the reference phase. The number of counts in the reference image is hence reduced by a factor of the number of phases leading to a reduced signal-to-noise ratio. The averaged uncorrected images served as intra-individual standard of reference in this study.

### Image quality assessment

The complete datasets of all twenty patients were provided to two experienced physicians: one radiologist and one nuclear medicine specialist, in the standard DICOM format. Both readers were blinded to the patient data and the used motion correction method. Image reading and rating was done in consensus, which also included the identification and localisation of the lesions. All patients were presented individually in random order and with a randomized order of the used correction method to avoid a potential bias in the scores.

The evaluation of the final PET images was performed using a software tool (syngo.via MM Oncology, Siemens Healthcare) that is targeted to oncology reading in hybrid modalities. An individual visibility score was determined for each detected lesion in each type of reconstructed image, and a single noise score per image was rated. Here, visibilities were rated on an ordinary integer scale ranging from 1 to 4 (1 = non-diagnostic, 2 = blurry, 3 = sharp, 4 = super sharp). The integer noise scale ranged from 1 to 3 (1 = high noise level, 2 = medium, 3 = low noise). Wilcoxon signed rank tests on the data were included to support the validity of all findings.

Furthermore, the maximum and mean standardized uptake values (SUVmax and SUVmean) of the individually determined lesion volumes were obtained. Therefore, isocontours at the threshold of 50% of SUVmax were used in a predefined search volume around the lesions. All mentioned scores and uptake values were furthermore obtained from a reference volume-of-interest (VOI) in the liver that was neither affected by a lesion nor showed significant uptake.

All motion-correction methods were compared in a pairwise fashion for each lesion using the obtained image ratings. Moreover, changes in the obtained lesion and reference SUVs were quantified and tested for significance by a Wilcoxon signed rank test.

## Results

**Fig 2:**
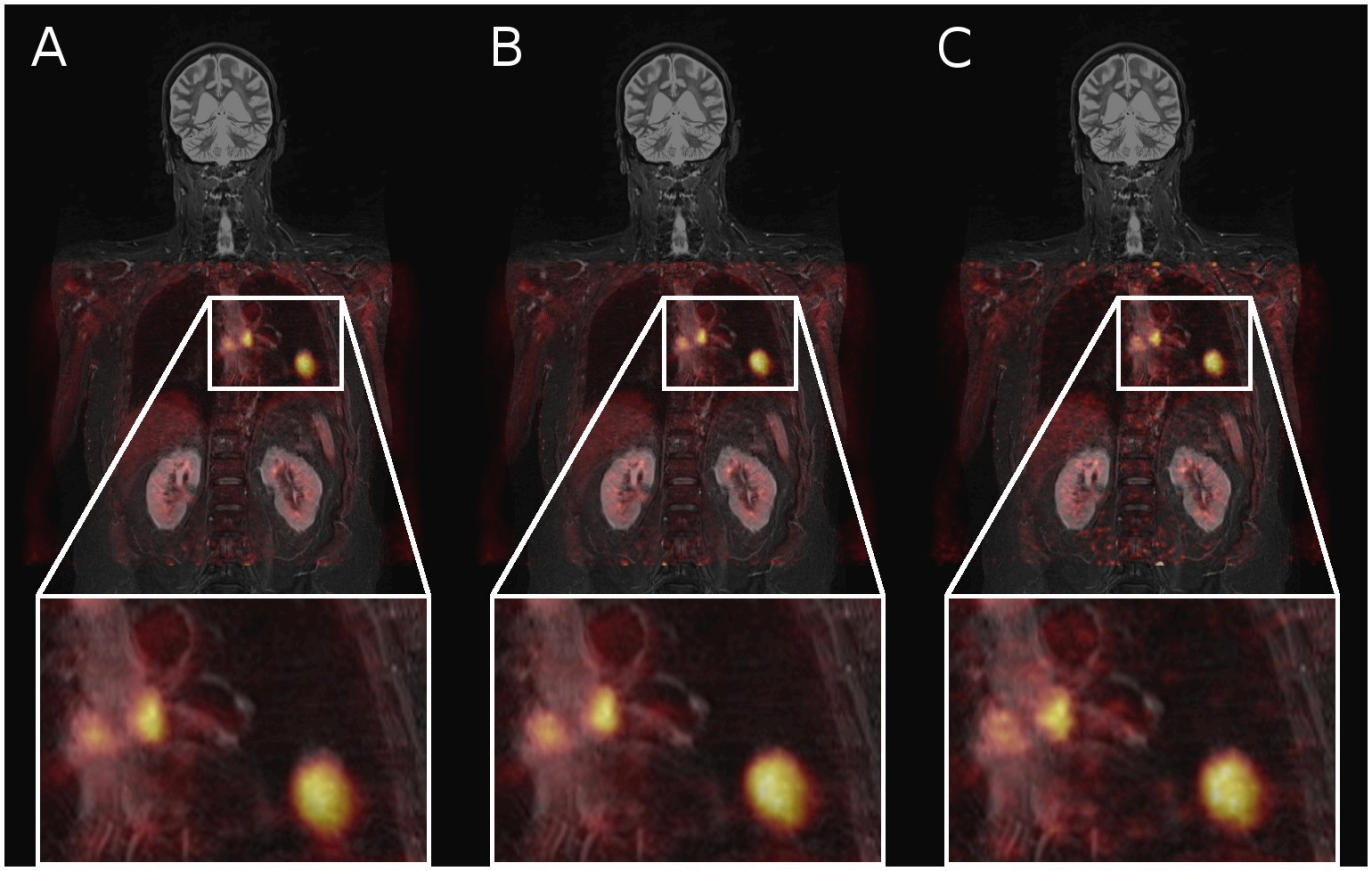
Merged MR and PET data of patient #17, who received the best MoCo image ratings, in a coronal view. PET data was reconstructed using (A) no motion correction, (B) the studied MoCo technique and (C) a conventional gating approach. Note the blurring of lesions in the uncorrected data (A) and the increased noise level in the gated PET image (C). MoCo received the highest scores in the visibility ratings of the three found lesions (3.7), while the uncorrected data was rated one mark less on average (3.0) and the gating approach having the worst scores (2.0). Noise scores of the MoCo and uncorrected images was rated similarly (3.0) while the gated method was rated worst (2.0).

**Fig 3:**
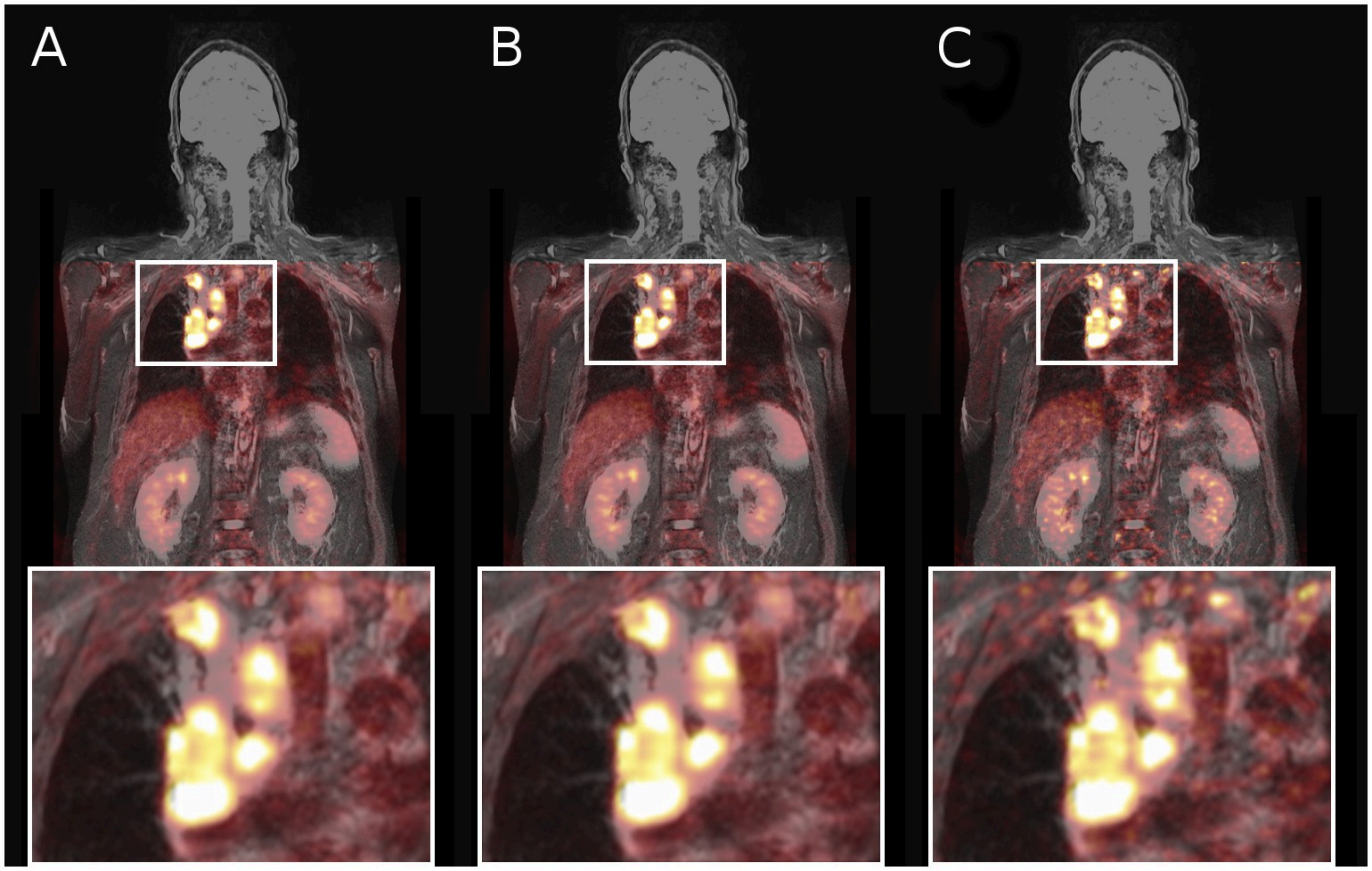
Merged MR and PET data of patient #19, with no improvement of image ratings for the studied MoCo reconstruction, in a coronal view. PET data was reconstructed using (A) no motion correction, (B) the studied MoCo technique and (C) a conventional gating approach. MoCo and static images received equal scores in both visibility and noise (3.0) of the found three lesions, whereas the gating approach showed lower scores for both, visibility (2.3) and noise (1.0).

**Fig 4:**
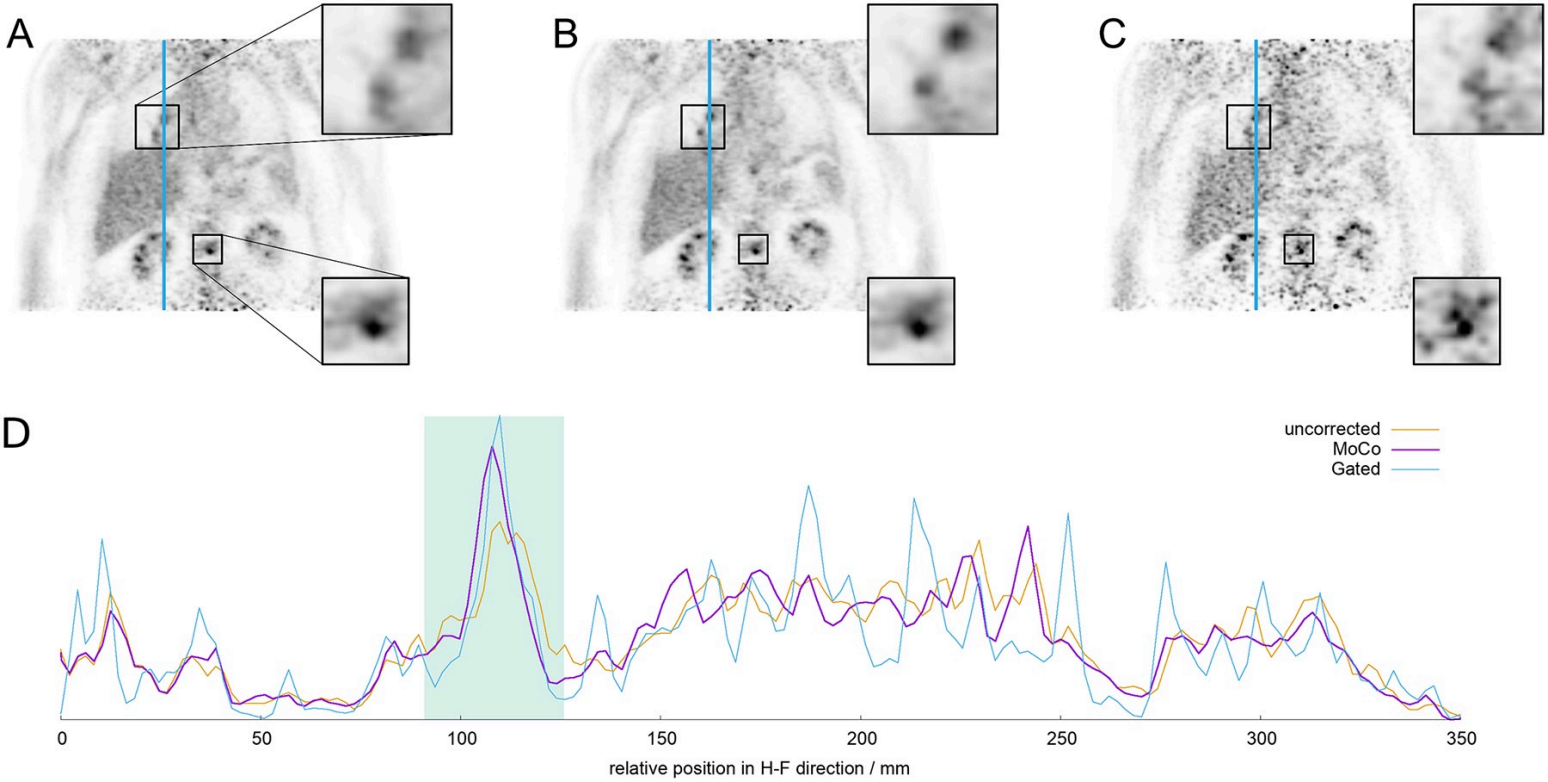
Visual comparison of PET images as obtained by uncorrected data (A), MoCo (B) and a gated reconstruction (C) of patient #15 containing a (motion-affected) lesion next to the hilum and a (static) osseous lesion in the lower spine. Zoomed regions of these lesions are shown in the small adjacent boxes. Note the enhanced sharpness and signal-to-noise ratio of the hilar lesion in the MoCo data in comparison to the other two methods, while the bone lesion in the region with less motion varies less between the uncorrected and MoCo reconstructions. The line profiles of the hilar lesion along the blue line in (A)-(C) for each method are depicted in (D). The green area denotes the position of the lesion. Within this region, average signal levels are comparable (MoCo vs. gated: 7.8%, MoCo vs. uncorrected: 0.6%), while the highest signal and standard deviation can be found in the gated images (maximum value 10.9% higher than in MoCo, standard deviation 13.3% increased). Linewidths (FWHM) of the lesion decrease from 12.2 mm (uncorrected) to 10.2 mm (MoCo) and 8.1 mm (gated), respectively.

All 20 patients were able to comply with study protocol, including breath holds during MRAC data acqusition, and limiting gross body movements throughout the PET acquisition. The implementation of the MoCo acquisition into the clinical protocol did not alter the general patient workflow, except extending the patient time in the scanner by the duration of the additional gradient spoiled echo acquisition for motion correction. A total of 43 lesions were found and evaluated in the patient datasets, leading to a total of 189 rater decisions (3 methods, 43 lesion visibilities and 20 image noise scores for each method). In the investigated patient cohort, the 43 lesions could be detected in all three motion correction techniques. Visual comparison of all methods revealed the expected improvement of the MoCo method over the uncorrected and gated approach in terms of sharpness and local noise levels (Figs 2, 3 and 4). However, neither of these enhancements led to a change in diagnosis, i.e. tumor gradings.

**Fig 5:**
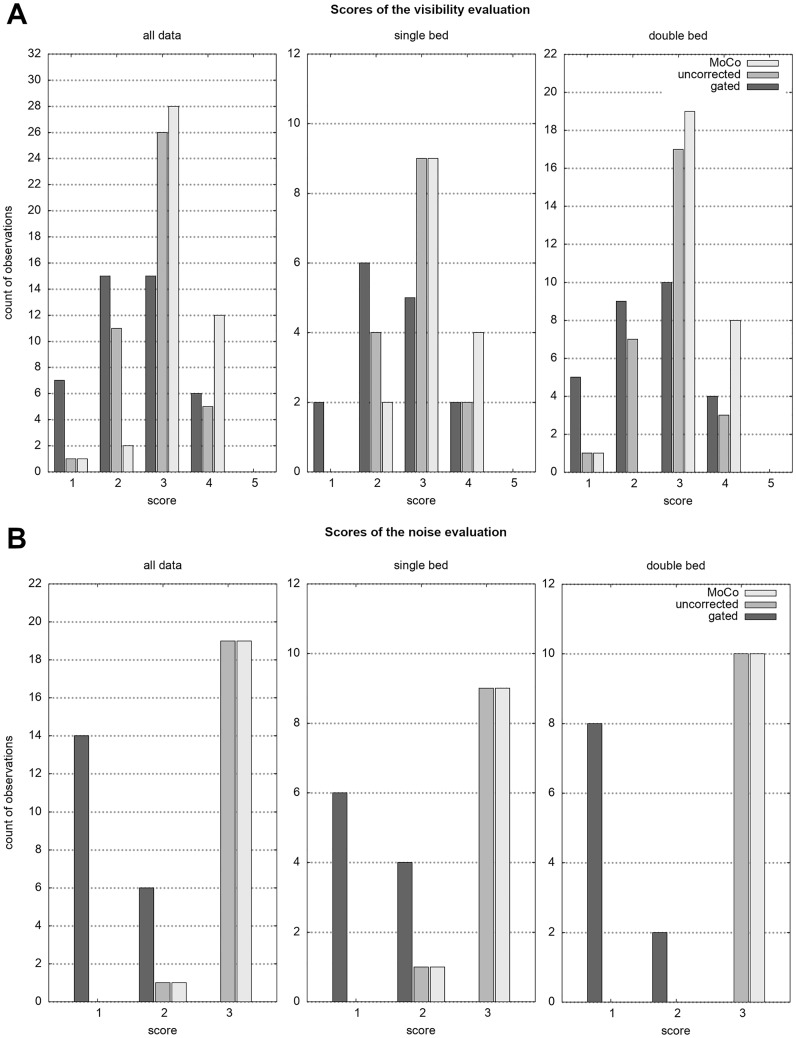
Box-Whisker plots comparing the visibility (A) and noise scores (B) in dependency of the inclusion of all data (white), the data of the ten patients with one bed position (light grey) and the data of the ten patients with two bed positions (dark grey). Note the equivalent scores of the intra-method comparison of patients, despite the different acquisition time of 5 and 10 minutes, respectively.

Average visibility scores (mean ± standard deviation) of 2.81 ± 0.66 (uncorrected), 3.19 ± 0.63 (MoCo) and 2.47 ± 0.93 (gated) were observed when including the complete patient data set. These values are 2.86 ± 0.64 (uncorrected), 3.13 ± 0.64 (MoCo) and 2.46 ± 0.96 (gated) when only considering the single-bed data, and 2.79 ± 0.69 (uncorrected), 3.21 ± 0.63 (MoCo) and 2.46 ± 0.96 (gated) when involving only the two-bed data, respectively (Fig 5). Thus, the different acquisition times per bed position (10 min for the single-bed acquisition vs. 5 mins for each of the two-bed acquisitions) did not introduce an impact on image quality scores. The same trend holds for the noise scores. On average, the PET images received noise ratings of 2.95 ± 0.22 (uncorrected and MoCo) and 1.30 ± 0.47 (gated). It should be noted that only one single noise rating deviated from the best mark (for MoCo and uncorrected). The majority of the noise scores for the gated method received the worst mark, regardless of the total duration of the PET acquisition (5 vs. 10 mins).

**Fig 6:**
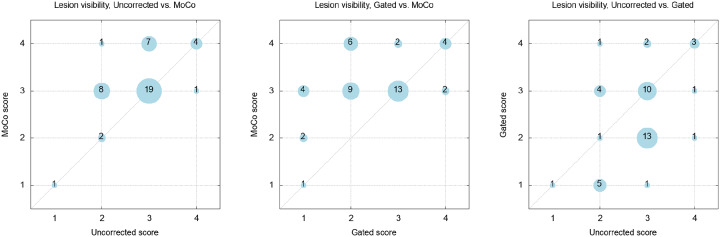
Bubble plots of the rating comparisons for the lesion visibility of all reconstruction methods. The number in the bubble denotes the number of occurrences of the respective rating pair. Note the tendency towards higher rating of the studied motion correction method (MoCo).

When comparing the visibility scores of the different motion correction reconstructions with each other it could be observed that for 42 cases the MoCo received similar (26 cases) or higher scores (16 cases) with respect to a simple time averaging using no motion correction (Fig 6). This trend still holds when relating the gated reconstruction to MoCo. Here, 23 cases with MoCo were rated better, while 18 cases got equal scores. The lesion visibility of completely uncorrected, yet averaged data was rated better than with conventional gating applied in the reconstruction.

**Fig 7:**
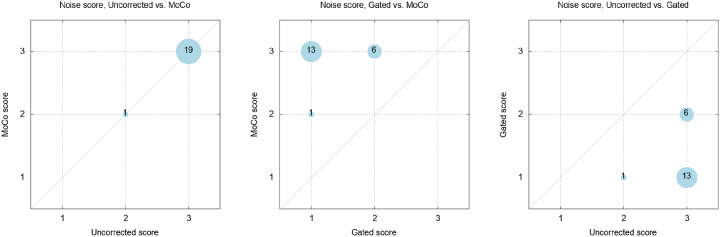
Bubble plots of the rating comparisons for the noise scores of all reconstruction methods. Whereas the uncorrected and MoCo data show equal ratings, the low statistical confidence of the gated data becomes evident.

Noise scores for MoCo and the uncorrected PET are identical. Further, as Fig 7 demonstrates, either one of these methods receives a better noise score than the corresponding images of the gated reconstruction, with a majority having the best achievable score, whereas the gated data was rated with the worst score, revealing the low count statistics of the latter.

As found by the Wilcoxon signed rank tests, each with two methods as samples, (Table 2), in particular the results from a comparison of MoCo to the gated and uncorrected approach indicate a significant change in the distribution of scores for visibility. Interestingly, the zero hypothesis could not be rejected (i.e. *p* > 0.05) for the comparison of noise scores of MoCo and the uncorrected approach, which is due the lack of difference in the underlying score distributions.

**Table 2:** Results of the Wilcoxon signed rank tests for the comparison between two reconstruction methods. Please note, that noise scores of the uncorrected vs. MoCo reconstruction are identical, thus yielding *p* = 1.

methods	category	*p*
gated vs. uncorr.	visibility	0.014
	noise	0.00005
gated vs. MoCo	visibility	0.00005
	noise	0.00005
uncorr. vs. MoCo	visibility	0.00035
	noise	1

**Fig 8:**
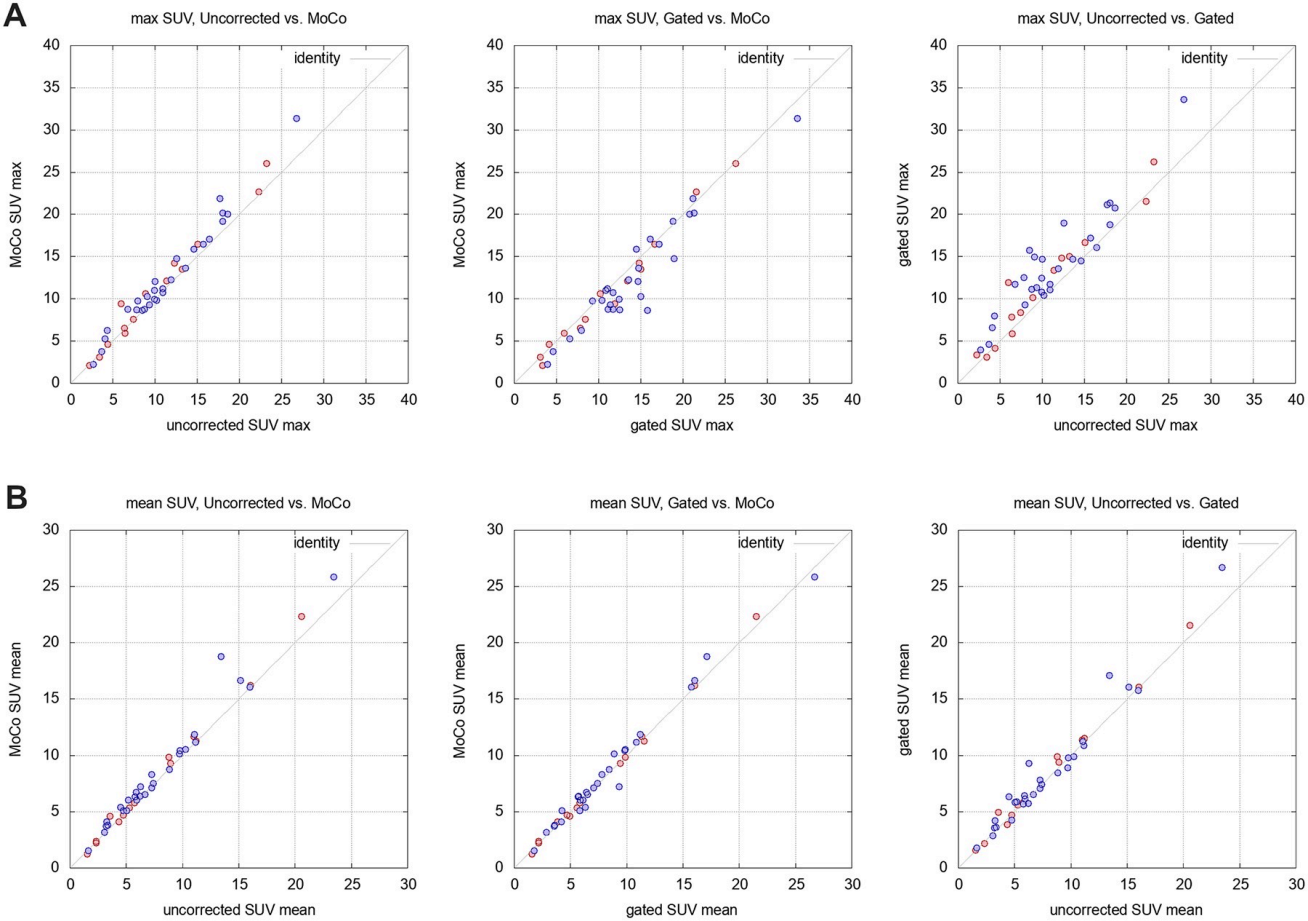
Comparison of SUVmax obtained by all three reconstruction methods. Red symbols depict the lesion values of the 10 min PET acquisition whereas purple symbols are associated with lesions that were found in the data of the two-bed acquisition with a 5 min acquisition per bed position.

All attenuation corrected SUVmax of the found lesions range from about 2 up to 34. SUVmax in the images of the gating approach are generally higher than their corresponding values in the MoCo and uncorrected data as shown in Fig 8. The average SUV deviations from a comparison of two reconstruction methods to each other are given in Table 3. When comparing to gated SUVmax, the MoCo SUVmax deviate on average by −1.19 ± 1.69 (equals -10.6%), while MoCo yields higher SUVmax (mean difference 1.02 ± 0.13, +9%) than the uncorrected method. On average, lesions in the gated images show a maximum standardized uptake value that is 2.21 ± 2.11, i.e. 25.4% higher than its corresponding value in the uncorrected data.

**Table 3:** Results of the comparison of the SUVs across reconstruction methods. The given deviations are average absolute differences of the first mentioned method with respect to the second. *p*-values were obtained from a pairwise Wilcoxon signed rank test and only calculated for the lesion-affected ROIs.

region	methods	ΔSUVmean	*p*	ΔSUVmax	*p*
lesion	MoCo vs. gated	0.14 ± 0.61	0.043	−1.19 ± 1.69	0.00004
	MoCo vs. uncorr.	0.56 ± 0.95	0.00001	1.02 ± 1.22	< 0.00001
	gated vs. uncorr.	0.42 ± 0.98	0.017	2.21 ± 2.11	0.00001
reference	MoCo vs. gated	−0.51 ± 0.30	-	−2.57 ± 1.06	-
	MoCo vs. uncorr.	0.02 ± 0.15	-	−0.07 ± 0.30	-
	gated vs. uncorr.	0.52 ± 0.33	-	2.50 ± 1.07	-

When comparing the mean SUVs of all methods to each other, a close distribution along the line of identity was observed (MoCo vs. uncorrected: mean deviation: 0.56 ± 0.95, 6.4%; gated vs. uncorrected: mean deviation: 0.42 ± 0.98, 5.9%; gated vs. MoCo: mean deviation: 0.14 ± 0.61, 1.2%). The best agreement of SUVmean was found for the comparison of MoCo with the gated reconstruction.

Consistently, the general behaviour of SUVmean and SUVmax in the lesions is reflected also in the SUVs of the reference volumes. Given the low uptake of the latter, the mean relative deviations are increased. Thus, the reference SUVmax that was obtained from the gated reconstruction was 2.57 ± 1.06 (vs. MoCo, relative increase: 91.3%) and 2.50 ± 1.07 units (vs. uncorrected data, relative increase: 86.0%) higher than its corresponding value in the respective other method. When comparing the MoCo approach with the uncorrected data, SUVmax of the MoCo were only slightly decreased by 1.5% in average (mean SUV decrease of 0.07 ± 0.30). These deviations dropped to 24.2% (increase gated vs. MoCo, 0.51 ± 0.30), 24.6% (increase gated vs. uncorrected, 0.52 ± 0.33) and -0.5% (decrease MoCo vs. uncorrected, 0.02 ± 0.15), respectively, when looking at the SUVmean of the reference regions. The diagnosis of all considered patient pathologies, i.e. tumor grading, therapy planning, etc. was not affected in any case.

## Discussion

In this study, a first evaluation of a newly implemented method for an MR-based motion correction of simultaneously acquired PET data into a clinical workflow is presented. While earlier studies [31, 33, 40] had a strong focus on a thorough technical analysis of the newly available motion-corrected image data and did not report about implications to the reader’s diagnosis, in this work the emphasis was rather on the integration of this method into a typical routine workflow and its assessment by experienced clinical readers.

The inherent spatial co-registration of the PET and MR data in a simultaneous hybrid PET/MR modality can be used to create high-resolution motion models of highly motion-affected regions such as the thorax. Hence, a mapping of the PET coincidences to the respective breathing phase derived from MR data can be used to retrospectively correct the acquired PET data involving all available data to eventually yield sharp and motion-free images of present lesions with high signal-to-noise ratios [17].

In comparison to the current reference-standards, i.e. no motion correction and a gating approach, where only data of a single motion state is included into the post-processing, the presented MoCo has the potential to use the best properties of both methods without the need of having any additional devices for motion-tracking attached to the patient. The use of the complete PET data rather than selecting only a low fraction (gating), leads to a lower perception of noise that is comparable the respective perception of the uncorrected PET images. Moreover, MoCo can lead to a notable increase of perceived contrast of the otherwise averaged data along the main breathing directions. Thus, small lesions and/or lesions with little uptake characteristics may benefit by the better sharpness and delineation at comparable count levels with the studied method. This also holds for patients with pathologies in the thorax, which is inherently affected by breathing motion. Due to the free-breathing acquisition of the MR data, the stress on these patients is reduced and overall image quality may improve in particular for those patients, who are unable to follow the breathing instructions. The protocol is extended by several minutes, but has potential to yield PET images with higher diagnostic quality.

When applying MoCo, an improved lesion visibility was confirmed in the thorough analysis by two experienced physicians. The enhanced lesion visibility in the MoCo data was reflected in the scores of the readers, which rated about one third of the lesions (38%) better than in the non-corrected images and more than half (56%) better than for the gated reconstruction. Furthermore, similar noise scores were achieved for MoCo and the uncorrected data, while the gated image data consistently yielded high noise levels, i.e. low noise ratings. This may be attributed to the reduced significance due to the much fewer considered counts that were taken into account in the image reconstruction. These findings support the results that have been demonstrated in terms of a technical image analysis by other studies [31, 33] and confirm the impact of the image improvement on the perception of the clinical readers. It is worth to be noted, that comparisons to the gated reconstruction are inherently dependent on the selected bin size and respective respiratory state. Thus, the reader ratings may shift to higher scores when optimizing these parameters to the best trade-off between residual motion and counts.

In this context, it shall be noted that in this study a rather short acquisition time of 5–10 minutes per bed position was used for collecting motion correction data. Thus, it is obvious, that gating significantly reduces the acquisition time per frame to only 1–2 minutes, increasing the noise levels accordingly. Gating, however may yield high diagnostic quality and low image noise in applications where PET data is sampled over rather long acquisition times as it is the case in dedicated thorax and cardiac imaging protocols (e.g. [41]).

Another finding of this study was a consistently elevated level of the gated SUVmax relative to their corresponding uncorrected and MoCo values, whereas this tendency is weaker for SUVmean. This may be attributed to the fact that the high noise levels of the gated data set introduce a rather high variance into the PET images that particularly has impact on SUVmax in a pre-defined volume-of-interest and leads to a comparably lower correlation to the other correction methods. Additionally, reduced motion blur in the MoCo and in the gated data leads to a local signal increase, i.e. higher SUVs, whereas in the averaged uncorrected images signal intensity is smeared out over a virtually larger lesion volume due to breathing motion. Since the evaluation of standardized uptake values is of particular interest when it comes to staging of lesions, the studied MoCo reconstruction may pave the way towards more accurate diagnosis in a routine clinical application of oncologic PET/MR. A larger dedicated patient cohort, however, may increase the significance of the findings of the present study.

Interestingly, no significant dependency of the visibility and noise scores on the duration of the motion model scan and the associated PET acquisition were found in accordance with [42]. However, the longer acquisition of 10 min per bed position yielded better count statistics for the determination of reliable and more stable SUVs for all methods and their correlations to each other. This is a straightforward finding, yet it suggests a suitable threshold for the minimal PET acquisition window as needed for both a reliable motion correction and images with high diagnostic value. It is worth to be noted, that throughout this study neither method led to a change of the diagnosed lesion malignancy or of the treatment of the patient.

Not least, the used MoCo reconstruction technique is not exclusively limited to oncology staging, but may also integrated into other clinical applications. As such, the MoCo technique may be applied as add-on to any whole-body PET/MR hybrid imaging protocol to provide improved lesion visibility in the one or two bed positions covering the thorax [27]. Furthermore, motion correction may provide a viable option for cardio-vascular applications, where e.g. the respiratory motion of the myocardium shall be reduced [43–45]. In particular, the potential of the application of motion-correction using the presented method using a radial stack-of-stars trajectory to reduce motion of the myocardium has been successfully demonstrated recently [46, 47].

## Conclusion

MR-based motion correction of PET data has proven to be a robust method in PET/MR to enhance image data that was acquired over several minutes in free breathing. The respiratory motion correction technique under study uses motion modelling and data binning to several respiratory states, which is based on a self-gated MR acquisition along with a subsequent warping of all PET data to a single reference state. The technique provides enhanced lesion visibilities along with a low noise level. This may support and facilitate the detection of lesions with PET/MR that are located in body regions affected by motion, such as the thorax and liver. Further in-depth studies with a larger patient cohort, however, may be required to estimate the impact on lesion staging.

## Supporting information

S1 Table Table with evaluation data from the clinicial readers. Scores and visibility rating are shown along with SUVmean and SUVmax for all lesions and one reference region ordered by the motion-correction approach.
(XLSX)
